# Community pharmacists' knowledge, attitudes, and practices regarding counselling on vitamins and dietary supplements in Malaysia: A study on complementary medicines

**DOI:** 10.1016/j.rcsop.2024.100410

**Published:** 2024-01-21

**Authors:** Rosamund Koo Wei Xin, Tan Wai Yee, Wong Zi Qin, Lau Kaiyee, Ali Haider Mohammed, Ali Blebil, Juman Dujaili, Bassam Abdulrasool Hassan, Angelina Lim

**Affiliations:** a School of Pharmacy, Monash University Malaysia, Jalan Lagoon Selatan, 47500 Bandar Sunway, Subang Jaya, Selangor, Malaysia; b Department of Pharmacy, Al Rafidain University College, Baghdad 10001, Iraq; c Swansea University Medical School, Swansea University, Swansea, UK; d Faculty of Pharmacy and Pharmaceutical Sciences Monash University, Victoria, Australia; e Murdoch Childrens Research Institute, Royal Children's Hospital, Victoria, Australia

**Keywords:** Vitamins, Dietary supplements, Pharmacy, Community, Complementary and alternative medicines

## Abstract

**Background:**

The utilization of vitamins and dietary supplements (DSs) among consumers in Malaysia has seen a notable increase. However, there is limited research available on how pharmacists in Eastern countries manage the provision of these products.

**Objective:**

This study aims to assess the knowledge, attitudes, and practices of community pharmacists in Malaysia regarding the provision of counselling services on vitamins and DSs. The findings will inform education strategies in this area.

**Methods:**

A cross-sectional quantitative study was conducted from February to April 2022 using a validated online-based questionnaire. The survey was distributed to community pharmacists across Malaysia through social media channels. *t*-test and ANOVA test were used for data analysis.

**Results:**

Among the 260 participants, 73.5% were categorized as having average product knowledge. Key concerns included a lack of knowledge about the indications of new products and when to discontinue their use. Regarding dosing in specific patient groups, 33.5% of pharmacists only occasionally consulted references and primarily relied on product labels. Furthermore, 29% of pharmacists believed it was unnecessary to refer patients to doctors when they experienced ongoing symptoms while taking vitamins or DSs. Interestingly, 44.6% of pharmacists believed there was a correlation between the efficacy of vitamins and their price, often recommending more expensive brands despite similar content.

**Conclusion:**

There is an opportunity to enhance the knowledge of pharmacists in Malaysia regarding vitamins and DSs. Education interventions should focus on areas such as dosing for specific patient groups, when to discontinue products, understanding new products, evidence-based efficacy of products for specific conditions, and providing a framework for appropriate referral to support pharmacists in their practice.

## 1 Introduction

Complementary and Alternative Medicines (CAMs) encompass a wide range of medical practices, products, and therapies that are not typically considered part of conventional (allopathic) medicine.^1^ Some studies have found that certain CAM practices can have synergistic effects when used with allopathic medicines, potentially enhancing the therapeutic outcome. For example, mindfulness meditation might improve the efficacy of antidepressant medications for some patients by reducing stress and promoting better adherence to medication regimes. In contrast, there is also evidence that some CAM practices can interact with allopathic medicines, sometimes in harmful ways. For instance, certain herbal supplements can interfere with the metabolism of drugs, leading to decreased efficacy or increased toxicity.^1^, ^2^, ^3^ Vitamins and dietary supplements (DSs), often incorporated within the framework of Complementary and Alternative Medicines (CAMs), command an essential role in the public health domain. These products are specifically designed to complement the diet and to serve as adjuncts in the prophylaxis and treatment of various health conditions. They are known to bolster the immune system and enhance metabolic functions.^4^ Additionally, they are utilized in the mitigation of risks associated with chronic diseases, contributing significantly to preventive healthcare measures.^5^ Available primarily through community pharmacies, these supplements necessitate informed counselling from pharmacists, who act as a critical interface between the products and the end consumers.^6^ The capability of pharmacists to offer comprehensive advice on DSs is impeded by gaps in their professional education and training. Many pharmacists rely on their undergraduate education, which may not cover the intricacies involved in DS counselling adequately.^7^, ^8^, ^9^ This issue is critical, as the contemporary healthcare landscape requires pharmacists to be well-versed in the interactions and contraindications of DSs, given the trend of consumer-led self-prescription.^8^

Empirical evidence from Iran has highlighted the inconsistency of pharmacists in providing vital information about DSs, suggesting a global need for improved educational resources and training.^10^^,^^11^ In the Malaysian context, the interplay between traditional health practices and modern medicine accentuates the need for pharmacists to be adept in guiding DS use. The country's regulatory environment for medicines is well-established, yet there remains a dearth of research exploring the role of community pharmacists in the realm of DSs.^8^ This situation is compounded by the cultural diversity within Malaysia, which influences health behaviors and practices, making the role of pharmacists even more complex and integral.^12^ Furthermore, the pattern of DS consumption in Malaysia indicates a high prevalence of use, which warrants targeted educational initiatives to ensure appropriate and safe use by the public.^13^^,^^14^

While research in Western nations has more extensively addressed the topic of pharmacists' roles in DS provision, similar studies in Eastern contexts like Malaysia are relatively scarce, underscoring a clear research need.^15^, ^16^, ^17^, ^18^, ^19^ This study is designed to investigate the knowledge, attitudes, and practices of pharmacists in Malaysia surrounding vitamins and DSs. It seeks to uncover educational gaps, propose enhancements to pharmacists' roles in DS advisement, and ultimately influence public health education to promote the safe use of DSs. The aspiration is to elevate the standard of DS advisement provided by pharmacists, which is expected to lead to improved health outcomes through more informed DS use by the Malaysian public.

## 2 Method

### 2.1 Study design and study population

A cross-sectional quantitative study was conducted using an online questionnaire. The study sample size was determined based on the population of community pharmacists in Malaysia, aiming for a representative sample. Participants were recruited on social media using snowball sampling. This technique was adapted for the online environment to reach the widespread community of pharmacists in Malaysia effectively. The process began with the identification of key contacts within the pharmacist community who were recognized as nodes of professional networks. .This technique was chosen due to the practical constraints of reaching a dispersed professional population and to utilize professional networks to ensure a broad reach. Online questionnaires were distributed using SurveyMonkey® to pharmacists in Malaysia who spoke English and who were Provisionally registered pharmacists (PRP), Fully registered pharmacists (FRP), or pharmacy assistants with a diploma in pharmacy, reflecting the composition of the community pharmacy workforce in Malaysia.

### 2.2 Survey instrument

Questionnaire items in this study were adapted from a few validated studies that assessed similar content [6, 10, 12, 13, 14]. The questionnaire, comprising 63 questions across four sections—demographics, knowledge, attitudes, and practice—was written in English and validated to ensure its reliability and applicability in the Malaysian context. Knowledge questions, designed as multiple-choice items, focused on common products sold in Malaysia. Attitude and practice questions utilized a five-point Likert scale to gauge the opinions and behaviors of community pharmacists.

To confirm the questionnaire's face and content validity, we conducted structured interviews with a representative panel of Malaysian community pharmacists. This panel evaluated the questions for relevance, clarity, and appropriateness, resulting in iterative modifications until consensus on content was achieved.

Furthermore, we employed a pilot study involving a separate cohort of pharmacists to test the questionnaire's reliability. Statistical analysis of the pilot study data yielded a Cronbach's alpha of 0.82 for the knowledge section, 0.89 for attitudes, and 0.85 for practice, indicating a high level of internal consistency for each domain. These values surpass the commonly accepted threshold of 0.70 for Cronbach's alpha, demonstrating the instrument's reliability.

In addition to face and content validity, we also ensured construct validity through exploratory factor analysis, which confirmed the questionnaire's ability to measure the constructs it was intended to. The factor loadings for all items were above the acceptable threshold of 0.50, providing further evidence of the instrument's construct validity.

The rigorous validation process we employed not only strengthens the reliability and validity of our questionnaire but also reinforces the credibility of the data collected, allowing for confident interpretation of the study results.

### 2.3 Data collection

The participants were recruited using social media, including Facebook, WhatsApp, Instagram, and Telegram, within a three-month period from 8 February 2022 to 30 April 2022. A sample size was calculated using the Raosoft calculator, reflecting the population of community pharmacists in Malaysia to be the required sample size of **385.** Besides, an official invitation was sent to the headquarters of chain pharmacies in Malaysia to disseminate the survey link among their community pharmacists as well as Malaysian community pharmacy groups in social media. Before filling out the surveys, participants were required to sign a formal consent form agreeing to participate in the study. To enhance the response rate and the representativeness of the sample, participants received weekly reminders to complete the survey. Participants were also asked to forward the online questionnaire to colleagues to complete.

### 2.4 Data analysis

Normality of variables was analysed and accepted with the Kolmogorov-Smirnov test at first. The hypotheses being tested through the *t*-test and ANOVA were focused on determining if there were statistically significant differences in the knowledge, attitudes, and practices (the dependent variables) between different groups of pharmacists categorized by their registration status and work experience (the independent variables). Independent *t*-test and ANOVA were used for inferential statistical analysis. A scoring system was implemented to measure the knowledge and attitudes of community pharmacists towards vitamins and DS based on expert panel discussions and previous literature studies.^20^, ^21^, ^22^, ^23^ The cut-off points for the classifications of knowledge, attitude, and practice categories were derived from percentile ranks based on descriptive statistics, ensuring a data-driven categorization. For the knowledge section consisting of 13 questions, one point was given for every correct response and zero for every inappropriate response. Participants' scores were then categorized: 0 to 5 as poor knowledge, 6 to 9 as average knowledge, and 10 to 13 as good knowledge. For the attitudes section consisting of 16 questions, one point was given for every appropriate response (strongly agree or agree for a positive statement and strongly disagree or disagree for a negative statement) and zero for every inappropriate or uncertain response.^20^ The final total score of attitude was classed, with a score of > 8 considered reflecting a positive attitude. For the practice section which consisted of 20 questions, one point was given for every appropriate response (never or rarely for a negative statement and often and always for a positive statement) and zero for every inappropriate or uncertain response. The final total score was classed, with the cut-off value of > 10 considered to reflect good practice towards vitamins and DS.^23^ Additionally, the distribution of the categories will be reported in the results section to provide a comprehensive overview of the data collected.

## 3 Results

### 3.1 Sociodemographic

Out of the 438 participants who were invited to participate in this survey, 260 participants (response rate = 59.36%) agreed to participate and completed the questionnaire. The majority of the participants were young adults with the mean age of 29.15 ± 5.849 years (Table 1). Most of them were female (61.2%) and majority of them were Chinese (73.1%). Majority of the participants had the highest qualification of Bachelor in pharmacy (87.3%) and approximately three quarters of the participants were fully registered pharmacists (FRP), with more than two-thirds of the participants graduating from private universities. Furthermore, 85.0% of the participants were full time pharmacists while 39 (15.0%) are part time or locum pharmacists, with 70.4% of them working in a chain pharmacy. Among the 260 participants, more than half of the participant pharmacists were working for 36 to 48 h weekly, with the majority (48.1%) having one to five years of working experience in the community setting. Approximately 51% of the participant pharmacists had frequent direct interactions with patients as shown in Table 1.

**Table 1:** Sociodemographic characteristics of participants (*N* = 260).

Characteristics	Categories	Frequency, n (%)
Gender	Male	101 (38.8)
	Female	159 (61.2)
Age group (29.15 ± 5.84)	21–35	234
	36–50	21
	> 50	5
Ethnicity	Malay	39 (15.0)
	Chinese	190 (73.1)
	Indian	22 (8.5)
	Others	9 (3.5)
Residence	Urban	233 (89.6)
	Rural	27 (10.4)
Highest Qualification	Diploma in pharmacy	8 (3.1)
	Bachelor in pharmacy	227 (87.3)
	Masters in pharmacy	25 (9.6)
	Other (please specify)	-
Current professional level	Provisionally Registered Pharmacist (PRP)	53 (20.4)
	Fully Registered Pharmacist (FRP)	198 (76.2)
	Not applicable	9 (3.5)
University	Public University	79 (30.4)
	Private University	181 (69.6)
Employment Contract Status	Full Time	221 (85.0)
	Part Time or Locum	39 (15.0)
Work Setting	Chain pharmacy	183 (70.4)
	Independent pharmacy	77 (29.6)
Working Hour (hours)	< 36	40 (15.4)
	36–48	143 (55.0)
	> 48	77 (29.6)
Experience (years)	< 1	65 (25.4)
	1–5	125 (48.1)
	> 5	69 (26.5)
Number of patients served daily	< 100	121 (46.5)
	100–200	107 (41.2)
	> 200	32 (12.3)
Direct interactions with patients	Never	0 (0.0)
	Rarely	2 (0.8)
	Sometimes	19 (7.3)
	Often	107 (41.2)
	Always	132 (50.8)

### 3.2 Knowledge

The majority of participants have average knowledge to counsel patients about vitamins and DSs (73.5%), while 21.2% have good knowledge and 5.4% have poor knowledge about vitamins and DSs (Table 2). When considering specific knowledge on common products, most of the pharmacists (83.2%) had a good understanding of the clinically proven benefits of common supplements including milk thistle, saw palmetto, ginkgo biloba, rooibos tea; however, most pharmacists were not familiar with indications of Eurycoma longifolia including treating minor illness, improving muscle strength and physical activities and reducing blood sugar level. More than half of the participants (52%) were also unaware that St John's wort should be stopped at least two weeks before scheduled surgery as it can lead to serious heart complications. Moreover, participants (24%) were also unsure about the dosage required for Labisia pumila, which is a popular herb used among females locally (Table 2).

**Table 2:** Summary of product knowledge questions (correct answers are flagged).

No.	Knowledge items	Frequency (%)
1	**There is no toxicity or side effects relating to large consumption of vitamins or supplements**	
	True	14 (5.4)
	False⁎	246 (94.6)
2	**Milk Thistle is recommended to treat hepatitis and cirrhosis**	
	True⁎	165 (63.5)
	False	95 (36.5)
3	**Dry hair or skin could indicate Vita**min **C deficiency**	
	True⁎	156 (60.0)
	False	104 (40.0)
4	**Saw palmetto is safe to be given to pregnant ladies with headaches, arthritis as a painkiller.**	
	True	10 (3.8)
	False⁎	250 (96.2)
5#	**Which one of the following supplements or medications can interact with digoxin? (choose all that apply)**	
	- St John's wort⁎	236 (90.8)
	- Antibiotics⁎	130 (50.0)
	- Coenzyme Q10	76 (29.2)
	- Omega-3	26 (10.0)
6	**Which statement best describes the evidence for *Ginkgo biloba*'s effects? (choose all that apply)**	
	- *Ginkgo biloba* helps improves blood circulation and heart health⁎	246 (94.6)
	- *Ginkgo biloba* help with cognitive functions and Alzheimer's disease⁎	217 (83.5)
	- *Ginkgo biloba* has anti-diabetic properties	30 (11.5)
	- *Ginkgo biloba* reduces the risk of getting stroke and cardiovascular diseases in elderlies	113 (43.5)
7#	**Which statements are correct for Labisia pumila (Kacip Fatimah)? (choose all that apply)**	
	- Labisia pumila can be given for pregnant or breastfeeding women	42 (16.2)
	- Labisia pumila 154 mg capsules can be taken twice daily⁎	82 (31.5)
	- Labisia pumila is used as health tonic for premenopausal and postmenopausal women⁎	234 (90.0)
	- Labisia pumila helps improve blood circulation	58 (22.3)
8	**What is the best time to take probiotics?** - 30 min before breakfast⁎ - Take after a heavy meal - 30 min after taking antibiotics - Before bedtime	218 (83.8) 24 (9.2) 9 (3.5) 9 (3.5)
9#	**Which of the following statements are correct for Eurycoma longifolia (Tongkat Ali)? (choose all that apply)**	
	- Eurycoma longifolia can treat minor illnesses such as ulcers, aches or fever⁎	32 (12.3)
	- Eurycoma longifolia can reduce blood sugar level⁎	38 (14.6)
	- Eurycoma longifolia can help regulate testosterone hormone in both men and women⁎	232 (89.2)
	- Eurycoma longifolia improve muscle strength and physical activities⁎	146 (56.2)
10#	**Which statement best describes the evidence for Rooibos tea's effects? (choose all that apply)**	
	- Rooibos tea contains caffeine similar to regular green tea or black tea	44 (16.9)
	- Rooibos tea contains antioxidant and can reduce LDL cholesterol⁎	171 (65.8)
	- Rooibos tea has anti-diabetic effects, improving insulin resistance and balance blood sugar level⁎	180 (69.2)
	- Rooibos tea can help with digestion and sleep	118 (45.4)
11#	**Which statement best describes the evidence for St. John Wort's effects? (choose all that apply)**	
	- St John wort can be taken concurrently for people with sleeping disorders who are taking sleeping pills at the same time	154 (16.9)
	- St John wort is used for mild to moderate depression, menopausal symptoms, and somatic symptom disorder⁎	203 (78.1)
	- St John wort should be stopped at least 2 weeks before scheduled surgery as it can lead to serious heart complications⁎	108 (41.5)
	- St John wort is a CYP3A4 enzyme inhibitor, thus should not be taken with drugs metabolised by CYP3A4 such as cyclosporine, diazepam, and erythromycin.	167 (64.2)
12#	**What do antioxidants do to our body? (choose all that apply)**	
	- Research shows that antioxidants protect cells against free radicals, to prevent heart disease, cancer etc.⁎	249 (95.8)
	- Antioxidant such as lutein can help prevent eye diseases⁎	165 (63.5)
	- Antioxidant supplements are not harmful to be taken in high dose	13 (5.0)
	- Antioxidant supplements will not interact and safe to be taken with other medications	25 (9.6)
13#	**Which statement best describes the evidence for omega-3's effects? (choose all that apply)**	
	- Omega-3 supplements reduce risk of heart diseases, cholesterol, improve cognition and relieve symptoms of arthritis⁎	246 (94.6)
	- Research shows that omega-3 supplements is beneficial for those with ADHD⁎	118 (45.4)
	- DHA improve brain function, while EPA dampen or prevent inflammation⁎	212 (81.5)
	- 500 mg omega-3 is used for general health to lower risk of coronary heart diseases, but more than higher intake of omega-3 of >1 g can be used for a range of additional heart conditions such as blood pressure, triglycerides ⁎	158 (60.8)
Level of knowledge (mean ± SD: 8.8677 ± 4.85)		n(%)
Poor knowledge		14 (5.4)
Average knowledge		191 (73.5)
Good Knowledge		55(21.2)

# participants were allowed to choose more than one options.

⁎ Correct Answer.

### 3.3 Attitudes

Table 3 shows the results of participants” overall attitude towards vitamins and DSs. Based on the participants' responses, about 80% of them agreed that supplements can bring a positive impact on public health, and are a major source of profit for community pharmacies. Nearly all participants (91.2%) believe pharmacists play an important role in advising patients regarding the use of DS, by helping patient in decision-making (34.3%) and providing appropriate counselling (91.2%). Most participants agreed you should frequently follow-up a patient after selling them a vitamin or DSs (60.8%), whereas 13.1% stated it was unnecessary to have frequent follow-ups with patients after their purchase. Nearly all the participants (91.2%) declared that they respected the choice of patient when choosing a vitamin and DSs, and 46.9% of the participants understood the desire for patients to take supplements to optimise their health. Some pharmacists (35%) even believe that it is safer for patients to take vitamins and DSs compared to taking conventional medications and around 32% of them claim that vitamins and DSs can replace certain medications such as cholesterol-lowering medications.

**Table 3:** Attitudes towards provision of sales of vitamins and DSs (N-260).

Attitude items	Strongly disagree n (%)	Disagree n (%)	Neutral n (%)	Agree n (%)	Strongly agree n (%)
I think supplements with vitamins have a positive impact on public health.	5 (1.9)	7 (2.7)	40 (15.4)	112 (43.1)	96 (36.9)
I think that vitamins and supplements are a major source of profit for community pharmacies.	5 (1.9)	7 (2.7)	40 (15.4)	112 (43.1)	96 (36.9)
I believe that vitamins and supplements should only be prescribed by dieticians or physicians.	52 (20.0)	95 (36.5)	65 (25.0)	34 (13.1)	14 (5.4)
I think that the price of vitamins and supplements is a major consideration when recommending them to the patients.	4 (1.5)	17 (6.5)	55 (21.2)	120 (46.2)	64 (24.6)
In my point of view, the patient's decision on health supplements are generally affected by the pharmacist's advice only.	16 (6.2)	73 (28.1)	84 (32.3)	77 (29.6)	10 (3.8)
I believe that there is correlation between the efficacy and the price of vitamins and supplements.	15 (5.8)	62 (23.8)	67 (25.8)	89 (34.2)	27 (10.4)
I think the laws and regulations regarding the practice of vitamins and supplements should be strengthened.	8 (3.1)	15 (5.8)	79 (30.4)	118 (45.4)	40 (15.4)
I agree that vitamins and dietary supplements can replace certain medications such as cholesterol-lowering medications.	53 (20.4)	73 (28.1)	51 (19.6)	67 (25.8)	16 (6.2)
I think it is necessary for pharmacists to be well prepared to educate patients about vitamins and supplements, such as discussing safety and efficacy information, and drug interactions, as well as information regarding their use in pregnancy and lactation, and comorbid conditions, as applicable.	4 (1.5)	3 (1.2)	16 (6.2)	98 (37.7)	139 (53.5)
I think it is not necessary to follow up with the patients on the use of their vitamins and supplements.	53 (20.4)	105 (40.4)	69 (26.2)	20 (7.7)	14 (5.4)
I feel burdensome when the patients repetitively consult me on the use of vitamins and supplements.	59 (22.7)	106 (40.8)	62 (23.8)	24 (9.2)	9 (3.5)
I agree that all vitamins and supplements are required to be taken on a regular basis in order to improve a patient's general health.	1 (0.4)	11 (4.2)	47 (18.1)	145 (55.8)	56 (21.5)
I think it is safer for patients to take vitamins and supplements to prevent or cure illnesses as compared to taking conventional medications	34 (13.1)	73 (28.1)	62 (23.8)	71 (27.3)	20 (7.7)
I prefer recommending vitamins and supplements with the highest profit despite having similar content as other brands.	32 (12.3)	48 (18.5)	88 (33.8)	61 (23.5)	31 (11.9)
I will respect the patients' decision if they do not purchase the vitamins and supplements that I have recommended.	2 (0.8)	4 (1.5)	17 (6.5)	126 (48.5)	111 (42.7)
I will continuously convince my patients to buy additional vitamins and supplements to help manage their general health.	16 (6.2)	33 (12.7)	89 (34.2)	92 (35.4)	30 (11.5)
Level of attitude	n(%)				
Negative	75 (28.8%)				
Positive	185 (71.2%)				

Approximately 44.6% of the pharmacists believed that there is a correlation between the efficacy and the price of vitamins and supplements, and around 35.4% (23.5% + 11.9%) of them preferred recommending more expensive brands despite having similar content as other brands.

### 3.4 Practice

Table 4 reports current practice of pharmacists and their provision of sales of vitamins and DSs. More than half of the participants (mean = 4.37/5) would always refer the patients and consumers to visit a doctor if their symptoms persist despite regular consumption of supplements, yet there are around 29% of the pharmacists who do not always refer the patients to the doctor. Many pharmacists also declared that they often or always educate patients and consumers about dosage and administration of vitamins and DSs (118, 45.4% and 109, 41.9% respectively). A gap in practice was seen (mean 3.58) in regards to consulting references for recommended doses for specific patient groups (e.g. pregnant women, renal patients) and 38 (10.8%) of the participants indicated that they rarely check the dose against a reference and usually go by the standard dose on the packaging.

**Table 4:** Practice of participant pharmacists in regards to provision of sales of vitamins and DSs.

Practice items	Never n (%)	Rarely n (%)	Sometimes n (%)	Often n (%)	Always n (%)	Mean
I collect the patient's or consumer's medical history before recommending vitamins and supplements.	4 (1.5)	8 (3.1)	55 (21.2)	105 (40.4)	88 (33.8)	4.02
I verify the indications of vitamins for prophylactic or therapeutic purposes if prescription is available.	3 (1.2)	6 (2.3)	43 (16.5)	118 (45.4)	90 (34.6)	4.1
I recommend vitamins and supplements very often without a doctor's prescription.	1 (0.4)	10 (3.8)	48 (18.5)	117 (45.0)	84 (32.3)	4.05
I have confidence in recommending and explaining the effectiveness and the safety of supplements.	1 (0.4)	7 (2.7)	60 (23.1)	122 (46.9)	70 (26.9)	3.97
I evaluate potential drug interactions with concomitant medications.	2 (0.8)	15 (5.8)	71 (27.3)	104 (40.0)	68 (26.2)	3.85
I counsel the patients and consumers about the side effects of vitamin consumption in large doses.	3 (1.2)	14 (5.4)	71 (27.3)	106 (40.8)	66 (25.4)	3.84
I review the patients' and consumers' allergy status to rule out contraindications of vitamins and supplements.	5 (1.9)	16 (6.2)	57 (21.9)	99 (38.1)	83 (31.9)	3.92
I consult references covering all RDA for infants, children, male, female, pregnant and lactating women, the elderly, in addition to renal and other chronic diseases when recommending vitamins and supplements.	6 (2.3)	32 (12.3)	71 (27.3)	107 (41.2)	44 (16.9)	3.58
I recheck the prescribed dose according to specific RDA before dispensing a prescription.	3 (1.2)	28 (10.8)	87 (33.5)	90 (34.6)	52 (20.0)	3.62
I evaluate the causes, signs and symptoms of vitamin deficiency to recommend the most suitable vitamin or supplement.	1 (0.4)	10 (3.8)	43 (16.5)	134 (51.5)	72 (27.7)	4.02
I am keen to follow up with the patients and consumers to monitor the effectiveness and side effects of the vitamins and supplements.	2 (0.8)	20 (7.7)	76 (29.2)	102 (39.2)	60 (23.1)	3.76
I counsel the patients and consumers about the benefits of natural sources of vitamins and supplements.	2 (0.8)	4 (1.5)	50 (19.2)	124 (47.7)	80 (30.8)	4.06
I educate patients and consumers about dosage and administration of vitamins and supplements.	1 (0.4)	5 (1.9)	27 (10.4)	118 (45.4)	109 (41.9)	4.27
I remind the patients and consumers to read specific usage instructions of vitamins and supplements.	1 (0.4)	18 (6.9)	47 (18.1)	107 (41.2)	87 (33.5)	4.00
I counsel the patients and consumers about several essential lifestyle issues such as adjusting food content and quantities to avoid vitamin toxicity, drinking an adequate amount of water, exercising regularly as well as smoking cessation.	1 (0.4)	13 (5.0)	40 (15.4)	103 (39.6)	103 (39.6)	4.13
I refer to valid web pages and scientific references before recommending vitamins and supplements.	1 (0.4)	15 (5.8)	62 (23.8)	102 (39.2)	80 (30.8)	3.94
I allocate enough time for providing advice to patients and consumers on vitamins and supplements.	1 (0.4)	13 (5.0)	59 (22.7)	124 (47.7)	63 (24.2)	3.90
I check the expiry date and content of vitamins and supplements before recommending them to patients and consumers.	2 (0.8)	10 (3.8)	41 (15.8)	96 (36.9)	111 (42.7)	4.17
I refer the patients and consumers to visit a doctor if their symptoms persist despite regular consumption of supplements.	3 (1.2)	9 (3.5)	17 (6.5)	92 (35.4)	139 (53.5)	4.37
I counsel the patients and consumers on the precautions and contraindications when consuming vitamins and supplements.	1 (0.4)	14 (5.4)	50 (19.2)	109 (41.9)	86 (33.1)	4.02

### 3.5 Sociodemographic factors and its impact on knowledge and attitudes

Table 5 explores whether sociodemographic factors of the participants had any impact on knowledge and attitudes. Pharmacists working in the chain pharmacies were shown to have a higher knowledge mean score compared to those working in independent pharmacies (*p* < 0.05). Pharmacists of Chinese ethnicity have a higher knowledge mean score (8.64) compared to other ethnicities. There is also a statistically significant difference between whether pharmacists had roles that predominately involved direct interaction with patients and knowledge mean score (*p* < 0.05).

**Table 5:** Analysis of difference between knowledge and attitude score with participants demographic characteristics.

Variables		Knowledge score mean	P	Attitude score mean	*P*-value @
Gender⁎	Male	8.23	0.066	9.13	**0.012**
	Female	8.61		10.04	
Residence⁎	Urban	8.50	0.305	9.72	0.594
	Rural	8.16		9.41	
University⁎	Public	8.28	0.235	9.11	**0.032**
	Private	8.55		9.93	
Employment contract status⁎	Full time	8.47	0.904	9.55	0.064
	Part time/ locum	8.44		10.46	
Work setting⁎	Chain pharmacy	8.60	**0.047**	9.80	0.324
	Independent pharmacy	8.15		9.42	
Age#	21–35	8.47	0.583	9.76	0.297
	36–50	8.30		9.24	
	>50	9.15		8.00	
Ethnicity#	Malay	7.90	**0.035**	8.90	0.236
	Chinese	8.64		9.79	
	Indians	8.06		10.27	
	Others	8.11		9.44	
Highest Qualification#	Diploma in pharmacy	7.69	0.104	7.125	**0.000**
	Bachelor in pharmacy	8.55		9.96	
	Masters in pharmacy	7.98		8.0	
Current professional level#	Provisionally Registered Pharmacist (PRP)	8.29	0.554	9.21	0.356
	Fully Registered Pharmacist (FRP)	8.53		9.79	
	Not applicable	8.17		10.22	
Working Hour (hours)#	< 36	8.36	0.811	10.15	0.422
	36–48	8.45		9.50	
	>48	8.56		9.78	
Experience (years)#	<1	8.34	0.57	9.61	0.902
	1–5	8.44		9.61	
	>5	8.63		9.77	
Number of patients served daily#	<100	8.33	0.356	9.80	0.715
	100–200	8.64		9.65	
	>200	8.38		9.34	
Direct interactions with patients#	Never	0	**0.032**	0	**0.003**
	Rarely	5.63		0	
	Sometimes	7.93		8.74	
	Often	8.46		9.54	
	Always	8.60		10.03	

⁎ T-test statistically significant.

# ANOVA test.

@ statistically significant <0.05.

Furthermore, there is no significant difference between residence setting, employment contract status, and work setting in terms of attitude score. Participants who graduated from private universities (9.93) having a better attitude towards providing counselling service to patients about vitamins and DS compared to those who graduated in a public university. (p < 0.05) (9.11). Female pharmacists also have a better score compared to male pharmacists (*p* = 0.012). There is also a significant difference between attitudes of pharmacists who directly interact with patients compared to those who don't (*p* = 0.0.03).

## 4 Discussion

Vitamins and DSs are used by an average of 20–30% of the population in developed countries.^24^ In Malaysia, 28.1% of the country's total adult population uses vitamin and mineral supplements.^25^ Compared to five years ago, customers expect more information about complementary medicines from their pharmacists.^9^ This study shows gaps in the lack of training on indications of new products, managing dosing of specific patients' groups (e.g. children, pregnant women), knowing when to stop medications, referral and follow-up, and bioequivalence of products. The study has also shown pharmacists in Malaysia aren't providing enough information on these products, and potentially not matching the growing expectations of the public. Further research should see whether the consumers in Malaysia are satisfied with the amount of information provided by the pharmacist to make an informed decision about the purchase, and whether they feel confident the dosing and safety information provided is sufficient.

Malaysian pharmacists generally have a positive attitude towards the provision of vitamins and dietary supplements (DSs). They acknowledge their role in ensuring safe use of these products. This aligns with findings from other studies, which report pharmacists' enthusiasm in advising on vitamins and DSs as part of their professional responsibilities.^11^^,^^26^^,^^27^ The favorable perspective is likely influenced by a common belief among Malaysians in the benefits of vitamins and DSs. Many Malaysians trust that these products can enhance general health. Additionally, some pharmacists perceive these supplements as safer alternatives to conventional medicines. In our study, 35% of participants expressed that it is safer for patients to use vitamins and DSs rather than conventional medicines. Moreover, about 32% believe that these products could substitute for certain medications, such as those used to lower cholesterol. Despite this, pharmacists tend to be skeptical about selling vitamins and DSs without solid scientific proof of their efficacy and safety. They show a negative attitude in such cases, as reflected in the literature.^8^^,^^28^

As there was a positive attitude shown from these participants, this could suggest educational interventions should be well-received by Malaysian pharmacists if they are accessible and help them with the supply of these products. More than half of the pharmacistss (80%) in this study recommended selling vitamins and DSs in pharmacies because it is seen as a major source of profit in community pharmacies. Furthermore, only more than half of the pharmacists had undergone formal education and training on vitamins and DSs. A systematic review conducted in Malaysia discussed that post-graduate pharmacists urged that an increasing DSs training should be increased to gain a comprehensive understanding of these products and to provide better counselling to inquiring patients.^29^ If there is a lack of evidence-based training, pharmacists have been shown to turn to media sources, including product advertisements to learn about the products.^30^ Lack of knowledge on these products has been shown to affect pharmacists' willingness to counsel patients on these products.^31^, ^32^, ^33^ Studies have also reported that pharmacists are generally less likely to educate patients about vitamins and herbal supplements.^21^^,^^34^ Future studies could integrate training on vitamins and DSs as part of renewal of registration which has shown to be successful in Singapore.^35^

An area of education that needs to be address is providing a framework for referral to support pharmacists when recommending vitamins and DSs. Some studies have found pharmacist will refer to doctor if the patient's symptoms persist despite regular consumption of supplements^36^^,^^37^ but not all our participants in our study encourage referral when needed, with 29% of pharmacists perceiving it to be unnecessary to refer to the doctor when patients have ongoing symptoms whilst taking vitamins or DSs. Concerns were also raised regarding the dosing for specific patient groups, including pregnant and patients with non-communicable disease groups. Many pharmacists, accounting for 33.5%, reported that they consult their reference materials only occasionally and typically rely on the product label for dosing information. This practice may be due to the inconvenience of making a proper referral or the belief that referrals are only necessary when the patient's condition becomes severe.^32^ Another gap was that results showed that community pharmacists were not consulting their references for dosing for specific patient groups and just counselling on the standard directions on the packaging. This could be due to limited reliable references, time constraints, and excessive workload, resulting in an insufficient allocation of time to patients.^21^^,^^38^ To better support pharmacists, there could be education on common patient groups and what to watch out for dosing, or a guide to where to best look for this information or online module or app, that provides the safest dose to give and to emphasis what toxicity signs to look out for in these patient groups.

When considering participant demographic factors, this study suggests that working at a chain pharmacy compared to an independent pharmacy, as well as having a more patient facing role, can significantly impact on the level of knowledge. This is consistent with a study conducted in China, where pharmacists at chain pharmacies had a better knowledge and attitudes towards pharmaceutical care and were able to practise more advanced pharmaceutical care than pharmacists at independent pharmacies.^39^ This could be because chain pharmacies tend to have more frequent product training and courses for their staff as compared to independent pharmacies, which can then affect their level of knowledge and quality of professional advice given to the patients. Moreover, community pharmacists who work in chain pharmacies provide more frequent one-on-one consultations for patients and deal with more sales of vitamins and DSs than independent pharmacies.^40^

This is the first study of its kind in Malaysia and will help inform policies that will help regulate these sales as these are popular products used by many Eastern countries. Further research could expand to other eastern countries to see what other facilitators and barriers to better provision of sales of CAMs exist. This study has some limitations; it has a modest sample size but has managed to capture a wide catchment of Malaysia and still provides a snapshot of the need for improvement in our services. The number of community pharmacists in Malaysia is estimated to be 2780 according to the Malaysian Pharmacist Society^41^; the participants in the study (*n* = 260) is approximately represents 10% of the population of pharmacists. The answers in this survey is self-reported and may not match to what these pharmacists actually do in real life and should be follow up by and observational study or mystery shopper or simulated patient study. It is a first step at investigating the main concerns and how training can be targeted.

Since COVID-19, the sales of vitamins and DSs, especially “immune boosting” supplements such as vitamin C, multivitamin, vitamin D, probiotics, selenium, zinc etc. increased significantly.^31^ It has been noted that the knowledge of community pharmacists selling vitamins and DSs is not increasing to match the increasing sales. Malaysian consumers also heavily rely on their pharmacist to provide accurate advice for these products.^42^ As vitamins and supplements are available online and in supermarkets, and with the surge of false advertisements of products to cure or prevent disease especially COVID-19 raised major concern in Canada, China, and the United States. [24]. Regulatory oversight and public education are vital for safe provision of these products and a pharmacist is in the best position to provide these services. Pharmacists should put extra effort in understanding the dosage, indications, drug-herb interactions, and when to stop herb products in certain circumstances for patient safety. There is a need for pharmacists to constantly update themselves about the newly available vitamins and DSs in the market, and update themselves. Consumers in Malaysia have a desire to use vitamins and DSs and its important future research investigates education interventions targeting dose in specific patient groups and when to stop products, new products, evidence-based efficacy of products for specific conditions, and provide a framework for appropriate referral to support pharmacists.

### 4.1 Limitation

This study has several limitations that should be acknowledged. Firstly, while efforts were made to achieve a representative sample of community pharmacists in Malaysia, the calculated sample size may have influenced result precision. Secondly, although rigorous validation procedures were employed for the survey questionnaire, self-reported data collection methods may introduce biases. Additionally, the recruitment approach via social media and snowball sampling may have led to self-selection bias. The cross-sectional design limits the study to a single point in time, hindering the assessment of temporal changes. Furthermore, the study's findings may not be entirely generalizable to regions with different healthcare systems and practices.

## 5 Conclusion

Our study has shown that while the majority of Malaysian pharmacists have an adequate understanding of vitamins and dietary supplements (DSs), there are knowledge gaps, particularly with less common products and dosing for specific patient groups. Attitudinally, pharmacists value their role in public health and patient counselling, yet some lack engagement in follow-up care. Practically, a notable portion does not always refer patients to doctors when needed and often defaults to label instructions for dosing.

These findings underscore the need for targeted educational interventions to deepen pharmacists' knowledge and refine their practices. By focusing on dosing, product cessation guidelines, and the efficacy of DSs, we can enhance the safe use of supplements and strengthen the referral process. This approach promises to elevate the standard of patient care provided by pharmacists in Malaysia.

## Ethics approval

Ethics approval was obtained from the Monash Human Research Ethics Committee (Ref. No: 2022–31,576).

## Funding

There was no funding for this work.

## CRediT authorship contribution statement

**Rosamund Koo Wei Xin:** Data curation, Formal analysis, Methodology, Project administration, Writing – original draft, Writing – review & editing. **Tan Wai Yee:** Data curation, Formal analysis, Methodology, Project administration, Software, Writing – original draft, Writing – review & editing. **Wong Zi Qin:** Data curation, Formal analysis, Methodology, Project administration, Software, Writing – original draft, Writing – review & editing. **Lau Kaiyee:** Data curation, Formal analysis, Methodology, Project administration, Software, Writing – original draft, Writing – review & editing. **Ali Haider Mohammed:** Conceptualization, Formal analysis, Investigation, Methodology, Resources, Software, Supervision, Validation, Visualization, Writing – review & editing. **Ali Blebil:** Methodology, Supervision, Writing – review & editing. **Juman Dujaili:** Methodology, Supervision, Writing – review & editing. **Bassam Abdulrasool Hassan:** Formal analysis, Methodology, Supervision, Writing – review & editing. **Angelina Lim:** Supervision, Writing – review & editing.

## Declaration of competing interest

The authors declare that they have no known competing financial interests or personal relationships that could have appeared to influence the work reported in this paper.

## References

[1] Tahir A.H., Tanveer M., Shahnaz G., Saqlain M., Ayub S., Ahmed A. (2023). Knowledge, attitude, and perceptions of healthcare professionals towards complementary and alternative medicine: a cross-sectional survey from twin cities of Pakistan. BMC Complement Med Therap.

[2] Guan A., Chen C.A. (2012). Integrating traditional practices into allopathic medicine. Columbia Univ J Glob Health.

[3] Tan G., Craine M.H., Bair M.J. (2007). Efficacy of selected complementary and alternative medicine interventions for chronic pain. J Rehabil Res Dev.

[4] Bayes J., Palencia J., Wardle J. (2023). Complementary and integrative medicine prevalence and utilization in international military and veteran settings and communities: a systematic review. Mil Med.

[5] Hathcock J. (2001). Dietary supplements: how they are used and regulated. J Nutr.

[6] El-Kadiki A., Sutton A.J. (2005). Role of multivitamins and mineral supplements in preventing infections in elderly people: systematic review and meta-analysis of randomised controlled trials. BMJ..

[7] Haque A.E., Abdullah M.N.A.B., Rahman N.S.B.A., Adzman S.A.B., Suhaimi W.H.A.B.W., Haque M. (2016). Assessment of knowledge, attitude and practices about nutritional supplements of the staff and students of universiti Kuala Lumpur-royal college of medicine Perak Malaysia. J Evolution Med Dent Sci.

[8] Waddington F., Naunton M., Kyle G., Thomas J., Cooper G., Waddington A. (2015). A systematic review of community pharmacist therapeutic knowledge of dietary supplements. Int J Clin Pharmacol.

[9] Braun L.A., Tiralongo E., Wilkinson J.M. (2010). Perceptions, use and attitudes of pharmacy customers on complementary medicines and pharmacy practice. BMC Complement Altern Med.

[10] Kwan D., Boon H.S., Hirschkorn K. (2008). Exploring consumer and pharmacist views on the professional role of the pharmacist with respect to natural health products: a study of focus groups. BMC Complement Altern Med.

[11] Alshahrani A. (2020). Knowledge, attitudes, and practice of community pharmacists towards providing counseling on vitamins, and nutritional supplements in Saudi Arabia. AIMS Public Health.

[12] Dabaghzadeh F., Hajjari R. (2018). Practice of community pharmacists related to multivitamin supplements: a simulated patient study in Iran. Int J Clin Pharmacol.

[13] Dores A.R., Peixoto M., Castro M. (2023). Knowledge and beliefs about herb/supplement consumption and herb/supplement–drug interactions among the general population, including healthcare professionals and pharmacists: a systematic review and guidelines for a smart decision system. Nutrients..

[14] Arumugam K. (2023).

[15] Tan M.L., Foong S.C., Foong W.C., Ho J.J. (2022). Use of Galactagogues in a multi-ethnic Community in Southeast Asia: a descriptive study. International. J Womens Health.

[16] Siti Z., Tahir A., Farah A.I. (2009). Use of traditional and complementary medicine in Malaysia: a baseline study. Complement Ther Med.

[17] Mohiuddin S.G., Aziz S., Iqbal M.Z. (2020). Knowledge, attitude, and practice of general population toward complementary and alternative medicines in relation to health and quality of life in Sungai Petani, Malaysia. J Pharm Bioallied Sci.

[18] Mohiuddin S.G., Aziz S., Ahmed R., Ghadzi S.M.S., Iqbal M.Z., Iqbal M.S. (2021). Use of traditional Chinese medicine in Malaysia: a knowledge and practice study among general population toward complementary and alternative medicine in relation to health and quality of life in Malaysia. J Pharm Bioallied Sci.

[19] Yusof H.M., Suffian N.A.S.M., Asma’ali K., Kamaruddin S., Noh M.F.M., Yusof A. (2023). Knowledge, Attitude and Practice on Complementary and Alternative Medicine (Cam) Associated With COVID-19. J Sustainabilit Sci Manag.

[20] Mehralian G., Yousefi N., Hashemian F., Maleksabet H. (2014). Knowledge, attitude and practice of pharmacists regarding dietary supplements: a community pharmacy-based survey in Tehran. Iranian J Pharmaceut Res: IJPR.

[21] Medhat M., Sabry N., Ashoush N. (2020). Knowledge, attitude and practice of community pharmacists towards nutrition counseling. Int J Clin Pharmacol.

[22] Alqahtani S.S., Alam N., Alharbi S.M. (2022). Knowledge, attitude, and practice regarding vitamin D and dietary supplements use: a community pharmacy based cross-sectional study in Jazan, Saudi Arabia. Curr Top Nutraceut Res.

[23] Emiru Y.K., Belay Y.B., Bizuneh G.K., Tegegn H.G. (2019). Community pharmacists’ knowledge, attitude, and professional practice behaviors towards dietary supplements: results from multi-center survey in Ethiopia. Nutr Diet Suppl.

[24] Sekhri K., Kaur K. (2014). Public knowledge, use and attitude toward multivitamin supplementation: a cross-sectional study among general public. Int J Appl Basic Med Res.

[25] Zaki N.M., Rasidi M.N., Awaluddin S.M., Hiong T.G., Ismail H., Nor N.M. (2018). Prevalence and characteristic of dietary supplement users in Malaysia: data from the Malaysian adult nutrition survey (MANS) 2014. Global J Health Sci.

[26] Lordan R. (2021). Dietary supplements and nutraceuticals market growth during the coronavirus pandemic–implications for consumers and regulatory oversight. PharmaNutrition..

[27] Bastani P., Jooybar M., Zadeh M.A., Samadbeik M. (2017). Community pharmacy-based survey on pharmacists’ knowledge, attitude, and performance regarding dietary supplements: evidence from south of Iran. National J Physiol, Pharm Pharmacol.

[28] Shilbayeh S.A. (2011). Exploring knowledge and attitudes towards counselling about vitamin supplements in Jordanian community pharmacies. Pharm Pract (Granada).

[29] Wong P.N., Braun L.A., Paraidathathu T. (2018). Exploring the interface between complementary medicine and community pharmacy in Malaysia—a survey of pharmacists. Malays J Public Health Med.

[30] Shraim N.Y., Shawahna R., Sorady M.A. (2017). Community pharmacists’ knowledge, practices and beliefs about complementary and alternative medicine in Palestine: a cross-sectional study. BMC Complement Altern Med.

[31] Ng J.Y., Tahir U., Dhaliwal S. (2021). Barriers, knowledge, and training related to pharmacists’ counselling on dietary and herbal supplements: a systematic review of qualitative studies. BMC Health Serv Res.

[32] Keely E., Tsang C., Liddy C., Farrell B., Power B., Way C. (2016). Rationale and model for integrating the pharmacist into the outpatient referral-consultation process. Can Fam Physician.

[33] Bukic J., Kuzmanic B., Rusic D. (2021). Community pharmacists’ use, perception and knowledge on dietary supplements: a cross sectional study. Pharm Pract (Granada).

[34] Culverhouse S.E., Wohlmuth H. (2012). Factors affecting pharmacists’ recommendation of complementary medicines–a qualitative pilot study of Australian pharmacists. BMC Complement Altern Med.

[35] Ang H.-G., Pua Y.-H., Subari N.A. (2013). Mandatory continuing professional education in pharmacy: the Singapore experience. Int J Clin Pharmacol.

[36] Ogbogu U., Necyk C. (2016). Community pharmacists’ views and practices regarding natural health products sold in community pharmacies. PloS One.

[37] Ooi G.S., Hassali A., Shafie A.A., Kong D., Mak V., Chua G.N. (2016). Assessment of community pharmacy services in Malaysia: perspectives from community pharmacists, general practitioners, consumers and health policy stakeholders. Value Health.

[38] Thandar Y., Mosam A., Botha J. (2019). Community pharmacists’ knowledge, attitude and practices towards the use of complementary and alternative medicines in Durban. South Africa Health SA Gesondheid.

[39] Xi X., Huang Y., Lu Q., Ung C.O.L., Hu H. (2019). Community pharmacists’ opinions and practice of pharmaceutical care at chain pharmacy and independent pharmacy in China. Int J Clin Pharmacol.

[40] Kho B.P., Hassali M.A., Lim C.J., Saleem F. (2017). Challenges in the management of community pharmacies in Malaysia. Pharm Pract (Granada).

[41] Liew C.H., Shabaruddin F.H., Dahlui M. (2022). The Burden of Out-of-Pocket Expenditure Related to Gynaecological Cancer in Malaysia. Healthcare.

[42] Hamidi N., Tan Y., Jawahir S., Tan E.H. (2021). Determinants of community pharmacy utilisation among the adult population in Malaysia: findings from the National Health and morbidity survey 2019. BMC Health Serv Res.

